# Association of the CALLY Index with Short-Term Treatment Response in Ankylosing Spondylitis

**DOI:** 10.3390/jcm15218446

**Published:** 2026-10-30

**Authors:** Bekir Torun, Ahmet Çiğiloğlu

**Affiliations:** 1 Department of Rheumatology, Necip Fazıl City Hospital, Kahramanmaraş 46080, Türkiye; 2 Department of Geriatrics, Necip Fazıl City Hospital, Kahramanmaraş 46080, Türkiye; cigiloglu@yahoo.com

**Keywords:** ankylosing spondylitis, CALLY index, treatment response, BASDAI, biomarker, inflammation

## Abstract

**Background/Objectives:** We examined the association between the pretreatment C-reactive protein–albumin–lymphocyte (CALLY) index and three-month clinical response to advanced therapy in patients with ankylosing spondylitis. **Methods:** We retrospectively analyzed 209 patients with ankylosing spondylitis who received biologic or targeted synthetic disease-modifying antirheumatic drugs. Laboratory measurements and Bath Ankylosing Spondylitis Disease Activity Index (BASDAI) scores were recorded at baseline; BASDAI was reassessed at three months. Response was defined as a reduction of at least 50% in BASDAI (BASDAI50). Analyses included Spearman’s rank correlation, multivariable logistic regression, nonlinear spline modeling, and receiver operating characteristic (ROC) analysis. **Results:** Among 209 patients, 167 (79.9%) achieved BASDAI50 response. Following recalculation from the recorded laboratory measurements, baseline CALLY was associated with treatment response, with a nonlinear exposure–response relationship (spline versus linear likelihood-ratio χ^2^ = 26.27, df = 2; *p* < 0.001; overall association *p* < 0.001). The CALLY AUC was 0.763 (95% bootstrap CI: 0.671–0.852), compared with 0.773 for CRP (paired bootstrap *p* = 0.485). The apparent and optimism-corrected AUCs of the adjusted spline model were 0.786 and 0.753, respectively. **Conclusions:** Baseline CALLY was nonlinearly associated with three-month BASDAI50 response and showed moderate discrimination between responders and nonresponders in this cohort. It did not demonstrate incremental value beyond CRP. Its clinical predictive utility remains unestablished and requires external validation.

## 1. Introduction

Ankylosing spondylitis (AS) is a chronic immune-mediated inflammatory disorder characterized predominantly by involvement of the spine and sacroiliac joints, often resulting in persistent pain, impaired mobility, and reduced quality of life [1,2,3]. Its development is influenced by a complex interplay of genetic and immunological factors, with HLA-B27-associated susceptibility, alterations in innate and adaptive immune responses, and inflammatory cytokine signaling contributing to disease pathogenesis [4,5,6,7]. Ongoing inflammation can promote structural changes in the axial skeleton and progressive functional limitations, underscoring the need for effective control of inflammatory disease activity [8,9].

The management of AS has evolved considerably following the availability of biologic and targeted synthetic disease-modifying antirheumatic drugs. Among these treatments, tumor necrosis factor and interleukin-17 inhibitors have shown substantial clinical benefits in patients with persistently active disease despite conventional therapeutic approaches [10,11,12]. However, therapeutic outcomes remain heterogeneous, and a considerable proportion of patients do not achieve a satisfactory clinical response. Real-world data also indicate that treatment outcomes may differ according to previous biologic exposure, emphasizing the heterogeneity of therapeutic response in AS [13]. Consequently, identifying accessible laboratory-based markers that may help distinguish patients according to their likelihood of achieving a favorable therapeutic response remains an important clinical objective.

C-reactive protein (CRP) and erythrocyte sedimentation rate (ESR) are routinely used to assess systemic inflammation in AS; however, neither fully reflects the complex biological processes underlying disease activity or therapeutic response. Recent research has consequently focused on composite and circulating biomarkers that integrate different aspects of systemic inflammation and host response. Several hematological inflammatory indices have been evaluated for monitoring disease activity and response to biological treatment in AS, although their relationship with clinical disease activity has not been consistent across studies [14]. Other blood-based biomarker approaches have similarly shown potential for identifying treatment responsive patient subgroups, supporting continued investigation of accessible pretreatment biomarker [15].

The C-reactive protein–albumin–lymphocyte (CALLY) index combines three routinely measured laboratory parameters: CRP, serum albumin concentration, and absolute lymphocyte count. This composite measure integrates laboratory findings related to systemic inflammation and host physiological responses, potentially offering a broader perspective than any single component considered separately. Although the CALLY index was originally explored primarily in patients with malignancies, its potential applications have subsequently been investigated in various inflammatory and immune-mediated disorders [16,17,18,19]. Nevertheless, its association with clinical outcomes in ankylosing spondylitis, particularly treatment response, has not been sufficiently characterized.

Against this background, we examined whether CALLY index values measured at baseline were associated with clinical improvement following three months of biologic or targeted synthetic therapy in patients with ankylosing spondylitis. We further explored the relationship between initial CALLY levels and changes in disease activity, as well as the discriminatory performance of this index in identifying patients who achieved a 50% reduction in the Bath Ankylosing Spondylitis Disease Activity Index (BASDAI50).

## 2. Materials and Methods

We conducted a single-center retrospective study at the Rheumatology Department of Kahramanmaraş Necip Fazıl City Hospital, Türkiye, evaluating adult patients diagnosed with AS according to the modified New York criteria. Medical records of patients treated between January 2024 and May 2025 were reviewed. Of these, 86 patients were excluded because of missing or incomplete serum albumin or CRP measurements. An additional 39 patients were excluded because of gastrointestinal disorders, including Crohn’s disease, ulcerative colitis, and celiac disease, which could independently influence inflammatory and nutritional parameters. Furthermore, 28 patients were excluded because of nonsteroidal anti-inflammatory drug use for osteoarthritis or the use of systemic corticosteroids or immunosuppressive agents for concomitant systemic diseases. The final analysis therefore included 209 patients. Three-month BASDAI assessments were available for all 209 included patients, with no missing follow-up BASDAI data in the final study population. The treatments evaluated were adalimumab (*n* = 59), etanercept (*n* = 46), golimumab (*n* = 34), infliximab (*n* = 26), certolizumab pegol (*n* = 20), secukinumab (*n* = 16), upadacitinib (*n* = 6), and tofacitinib (*n* = 2). All patients receiving TNF inhibitors or secukinumab were biologic-naive at treatment initiation. The eight patients receiving JAK inhibitors had previously been treated with TNF inhibitors. Baseline laboratory measurements, including CRP, serum albumin, and absolute lymphocyte count, and BASDAI assessments were obtained immediately before initiation of the treatment evaluated in this study. Follow-up BASDAI assessments were performed approximately three months after treatment initiation, according to routine outpatient appointment schedules.

The patient screening and selection process, including the reasons for exclusion, is summarized in Figure 1.

Research-related extraction and retrospective review of medical records were undertaken only after ethics committee approval on 20 October 2025 (approval No. 286).

Patient demographics, therapeutic regimens, laboratory results, and clinical disease activity measures were retrieved from electronic medical records. The laboratory variables recorded at baseline comprised CRP, ESR, albumin, hemoglobin, total leukocyte count, platelet count, and absolute lymphocyte count. For each patient, the CALLY index was determined using the following Equation:

CALLY index = [Serum albumin (g/dL) × Absolute lymphocyte count (cells/μL)]/[CRP (mg/dL) × 10^4^]

No patients had CRP values equal to zero or below the laboratory detection limit; therefore, no substitution or adjustment of CRP values was required.

Serum CRP was quantified by nephelometry (analytical detection limit, 0.3 mg/L; institutional reference upper limit, 5 mg/L). Albumin was determined photometrically (reference interval, 3.5–5.2 g/dL), and absolute lymphocyte counts were measured by fluorescence flow cytometry (reference interval, 1.2–3.4 × 10^3^/µL). CRP measurements in mg/L were converted to mg/dL for CALLY calculation.

Disease activity was evaluated at treatment initiation and again after three months using the BASDAI. This patient-reported instrument consists of six questions covering fatigue, spinal pain, peripheral joint manifestations, areas of localized tenderness, and both the intensity and duration of morning stiffness. Responses to each question are rated on a numerical scale ranging from 0 to 10. The two morning-stiffness items are averaged, and the resulting value is combined with the first four item scores to calculate the final BASDAI score. Treatment response was defined according to BASDAI50, with patients achieving a reduction of at least 50% from baseline classified as responders and the remaining patients as nonresponders. Percentage improvement in BASDAI was additionally calculated as [(baseline BASDAI − month-3 BASDAI)/baseline BASDAI] × 100.

Continuous data were described using means and standard deviations or medians with interquartile ranges, depending on their distribution, whereas categorical data were expressed as frequencies and percentages. Differences between patients who achieved BASDAI50 and those who did not were examined using Student’s independent-samples *t*-test, Welch’s *t*-test, or the Mann-Whitney U test, according to the characteristics of the data. Pearson’s chi-square test was applied to categorical comparisons when the required assumptions were met.

Relationships between baseline laboratory measurements and the percentage change in BASDAI were investigated using Spearman’s rank correlation coefficient. To determine whether baseline CALLY was independently associated with BASDAI50 achievement, a multivariable binary logistic regression model was fitted with age, sex, and baseline BASDAI included as covariates. Effect estimates were presented as odds ratios (ORs) accompanied by their 95% confidence intervals (CIs). Age and sex were included as demographic covariates, while baseline BASDAI was included to account for differences in disease activity before treatment initiation. The number of covariates was deliberately limited in view of the 42 patients who did not achieve BASDAI50 response, to reduce the risk of model overfitting. To investigate whether the direction of the association between baseline CRP and BASDAI50 response was consistent within a clinically more homogeneous treatment group, an exploratory subgroup analysis was performed among patients receiving TNF inhibitors. The association was evaluated using multivariable logistic regression adjusted for age, sex, and baseline BASDAI. The primary analysis focused on the association between baseline CALLY and BASDAI50 response at month 3. Correlation analyses involving individual laboratory parameters and additional subgroup analyses were considered exploratory. Given the multiple statistical comparisons performed, findings from exploratory analyses were interpreted cautiously, particularly when *p*-values were close to the conventional significance threshold. No formal adjustment for multiple comparisons was applied.

The discriminatory performance of baseline CALLY for distinguishing BASDAI50 responders from nonresponders was assessed through receiver operating characteristic (ROC) curve analysis. The area under the ROC curve (AUC) was estimated with its corresponding 95% CI, and the threshold maximizing the Youden index was selected as the optimal cutoff. The linearity assumption for baseline CALLY in the multivariable logistic regression model was assessed by comparing the primary model with a model incorporating a cubic B-spline function of CALLY. Both models were adjusted for age, sex, and baseline BASDAI. Model fit was compared using a likelihood-ratio test. A sensitivity analysis was also conducted after excluding patients receiving JAK inhibitors who had previously been exposed to TNF inhibitors.

For nonlinear modeling, a cubic B-spline of CALLY with three degrees of freedom and boundary knots at the observed minimum and maximum was fitted, with adjustment for age, sex, and baseline BASDAI. Likelihood-ratio tests assessed departure from linearity (2 df) and overall CALLY association (3 df). Adjusted predicted response probabilities were estimated with 95% confidence intervals. CRP-only, CALLY-only, and combined CRP–CALLY logistic models used linear biomarker terms with the same covariates; the combined model was exploratory because CALLY contains CRP mathematically. ROC AUCs were compared by 5000 paired, outcome-stratified bootstrap resamples; bootstrap percentile intervals and a two-sided sign-based *p*-value were calculated. The complete spline-model fitting procedure was internally validated using 600 bootstrap resamples, estimating optimism-corrected AUC, Brier score, and calibration slope. Additional analyses were conducted using Python (version 3.13.5) with statsmodels (version 0.14.6), patsy (version 1.0.2), and scikit-learn (version 1.8.0).

The variability of the Youden-optimal CALLY threshold was assessed using 5000 outcome-stratified bootstrap resamples, with the threshold re-estimated in each resample. The bootstrap median and 2.5th–97.5th percentile interval were summarized separately from the optimism-corrected performance estimates.

Statistical significance was evaluated using two-tailed tests, with a *p*-value below 0.05 considered significant. Statistical analyses were performed using IBM SPSS Statistics, version 27.0 (IBM Corp., Armonk, NY, USA).

## 3. Results

The final study population comprised 209 patients, of whom 167 (79.9%) achieved BASDAI50 at the three-month assessment and 42 (20.1%) did not. In the overall study population, mean BASDAI decreased from 6.50 ± 0.79 at baseline to 2.73 ± 0.90 at month 3. Among responders, mean BASDAI decreased from 6.49 ± 0.77 to 2.39 ± 0.53, corresponding to a mean percentage improvement of 63.08 ± 7.23%. In nonresponders, mean BASDAI decreased from 6.55 ± 0.86 to 4.11 ± 0.74, with a mean percentage improvement of 37.07 ± 9.65%. The mean absolute reduction in BASDAI was 4.10 ± 0.72 among responders and 2.44 ± 0.76 among nonresponders. The responder and nonresponder groups were comparable in terms of age, sex, and initial BASDAI scores, with no statistically significant differences observed (all *p* > 0.05). Median baseline CALLY values were significantly greater among responders than nonresponders [1.12 (0.54–2.75) versus 0.32 (0.16–0.81), *p* < 0.001]. In contrast, responders exhibited lower pretreatment CRP concentrations [0.92 (0.33–1.65) versus 2.89 (0.98–4.38) mg/dL, *p* < 0.001] and ESR values [19.0 (10.0–32.0) versus 32.5 (17.65–55.0) mm/h, *p* < 0.001]. Hemoglobin concentrations were higher in the responder group (14.11 ± 1.76 versus 13.25 ± 2.08 g/dL, *p* = 0.007), while platelet counts were lower (286.06 ± 70.63 versus 326.48 ± 106.49 × 10^3^/µL, *p* = 0.024). Lymphocyte counts, total leukocyte counts, and serum albumin concentrations did not differ significantly between the two groups (Table S1).

Spearman’s rank correlation analysis demonstrated a positive association between baseline CALLY and the percentage reduction in BASDAI (ρ = 0.316, *p* < 0.001). Negative correlations were identified for CRP (ρ = −0.314, *p* < 0.001) and ESR (ρ = −0.243, *p* < 0.001). Hemoglobin showed a positive correlation with percentage improvement in BASDAI (Spearman’s ρ = 0.204, *p* = 0.003). A weaker positive correlation was observed for lymphocyte count (Spearman’s ρ = 0.138, *p* = 0.047); however, this exploratory finding should be interpreted cautiously in view of the multiple comparisons performed. Conversely, the correlations involving serum albumin (ρ = 0.087, *p* = 0.212) and platelet count (ρ = −0.132, *p* = 0.057) did not reach statistical significance (Table S2).

In the covariate-adjusted linear logistic model, the CALLY odds ratio was 1.925 per one-unit increase (95% CI: 1.297–2.857; *p* = 0.001). This linear effect estimate is not constant across the observed CALLY range: a cubic B-spline provided significantly better fit than the linear model (likelihood-ratio χ^2^ = 26.27, df = 2; *p* < 0.001). The overall adjusted association was significant (likelihood-ratio χ^2^ = 41.96, df = 3; *p* < 0.001). Excluding eight JAK-inhibitor recipients yielded a linear adjusted OR of 1.790 (95% CI: 1.217–2.632; *p* = 0.003; *n* = 201). The adjusted spline response curve with 95% confidence intervals is presented in Figure 2.

In the TNF-inhibitor subgroup (*n* = 185), higher baseline CRP was inversely associated with BASDAI50 response after adjustment for age, sex, and baseline BASDAI (OR per 1 mg/dL = 0.445, 95% CI: 0.327–0.604; *p* < 0.001). By treatment class, BASDAI50 response occurred in 148 of 185 TNF-inhibitor recipients, 15 of 16 secukinumab recipients, and 4 of 8 JAK-inhibitor recipients. These subgroup findings were exploratory.

Baseline CALLY had an apparent ROC AUC of 0.763 (95% bootstrap CI: 0.671–0.852); the corresponding CRP AUC was 0.773 (95% bootstrap CI: 0.685–0.858). In 5000 paired bootstrap resamples, the CRP–CALLY AUC difference was 0.010 (95% CI: −0.018 to 0.038; *p* = 0.485). The Youden-optimal CALLY threshold was 0.358 (sensitivity 87.4%, 95% Wilson CI approximately 81.5–91.6%; specificity 59.5%, 95% Wilson CI approximately 44.5–73.0%; 146 true positives, 21 false negatives, 25 true negatives, and 17 false positives; Table S4, Figure 3). The bootstrap median threshold was 0.358 (95% percentile interval 0.235–0.788). Adding CALLY to a CRP-containing covariate-adjusted linear logistic model did not significantly improve model fit (likelihood-ratio *p* = 0.350). The apparent AUC of the adjusted spline model was 0.786; after 600 bootstrap resamples, the optimism-corrected AUC was 0.753, the corrected Brier score was 0.130, and the corrected calibration slope was 0.853. These results represent internal validation, not external clinical validation.

## 4. Discussion

Identifying readily available markers associated with treatment response remains an important issue in the management of ankylosing spondylitis. Despite their widespread use as indicators of systemic inflammation, CRP and ESR do not fully reflect the extent of clinical improvement in ankylosing spondylitis. Moreover, changes in patient-reported disease activity may occur independently of measurable alterations in inflammatory markers. This has increased interest in biomarkers that may provide additional information on disease activity and treatment response [20,21]. Our findings demonstrated that patients with higher CALLY index values at baseline were more likely to achieve a BASDAI50 response after three months of treatment. Furthermore, baseline CALLY showed a positive correlation with the magnitude of BASDAI improvement. This association remained statistically significant in the multivariable analysis accounting for age, sex, and initial disease activity measured by BASDAI. Its AUC of 0.763 further indicated a moderate ability to discriminate responders from nonresponders. Taken together, our findings support a possible role for CALLY as a simple laboratory-based marker associated with short-term clinical response, while also indicating that its value should be considered together with established inflammatory markers rather than as a replacement for them.

CALLY incorporates CRP, albumin, and lymphocyte count and therefore combines inflammatory, nutritional, and immune-related laboratory parameters. Routine hematological parameters have also been used to construct composite indices for evaluating inflammatory activity. Previous investigations in ankylosing spondylitis have reported associations between disease activity and indices such as the NLR, PLR, and SII. Furthermore, reductions in some of these markers following tumor necrosis factor inhibitor therapy have been described, suggesting a potential relationship with therapeutic response [21]. An important practical feature of these indices is their reliance on laboratory measurements already obtained during routine clinical care, eliminating the need for additional investigations. Although albumin and lymphocyte count contribute to the CALLY index, these parameters are influenced by multiple physiological and pathological factors and should not be interpreted as direct measures of nutritional status or immune competence without additional clinical assessment. Unlike NLR, PLR, and SII, the CALLY index incorporates CRP, albumin, and lymphocyte count into a single measure. However, its predictive performance was not directly compared with these hematological indices in the present study; therefore, no superiority over these established indices can be inferred.

Our findings should, however, be interpreted together with conventional inflammatory markers. Patients who achieved BASDAI50 exhibited significantly lower CRP and ESR values at baseline compared with nonresponders. In contrast, neither serum albumin concentrations nor absolute lymphocyte counts showed statistically significant differences between the two groups. Previous studies involving patients with ankylosing spondylitis and axial spondyloarthritis have identified elevated pretreatment CRP concentrations and other markers of inflammatory activity as factors associated with a greater likelihood of responding to tumor necrosis factor inhibitor therapy [22,23,24,25,26]. Therefore, our results do not indicate that CALLY is superior to CRP. An important finding of the present study was the association between lower baseline CRP concentrations and a greater likelihood of achieving BASDAI50 response. This contrasts with previous studies reporting that elevated baseline CRP is associated with a more favorable response to TNF inhibitor therapy in axial spondyloarthritis. Several factors may contribute to this discrepancy, including differences in patient selection, previous treatment exposure, treatment regimens, and the timing of baseline assessments. However, these explanations remain speculative and cannot be established from the present retrospective data. Notably, the inverse association between baseline CRP and BASDAI50 response persisted in an exploratory analysis restricted to patients receiving TNF inhibitors. Therefore, treatment-class heterogeneity alone does not appear to explain this finding. Differences in study populations and outcome definitions may also contribute, particularly because BASDAI50 reflects patient-reported symptom improvement rather than objective suppression of inflammation. These findings require confirmation in independent cohorts. In exploratory analyses, CALLY did not outperform CRP (AUC 0.763 versus 0.773), and adding CALLY to a CRP-containing regression model did not significantly improve model fit (*p* = 0.350). The corresponding Spearman coefficients were ρ = −0.314 for CRP and ρ = 0.316 for CALLY. Although CALLY can be calculated from routinely available laboratory parameters without additional testing, our findings do not demonstrate a clinically meaningful predictive advantage over CRP alone. Therefore, routine calculation of CALLY specifically for treatment-response prediction cannot currently be recommended. Its potential value as a complementary composite marker requires further prospective evaluation.

The search for treatment-response biomarkers in axSpA has extended beyond routine inflammatory markers. Complement activation, intestinal barrier-related biomarkers, circulating cell-free DNA, and molecular immune profiles have all been investigated in relation to disease activity or therapeutic response [27,28,29,30]. Baseline complement activation markers have shown associations with subsequent TNF inhibitor response, while LBP and calprotectin have been associated with disease activity and changes during treatment [27,28]. Circulating cell-free DNA and molecular immune signatures have also shown associations with treatment response [29,30]. Taken together, these observations underscore the multifactorial nature of treatment response in axial spondyloarthritis and indicate that no individual laboratory marker is likely to adequately reflect the diverse biological processes influencing therapeutic outcomes.

We used BASDAI50 to define clinical response at month 3. BASDAI50 has been used as a major clinical response criterion in studies evaluating response to TNF inhibitors in AS [31]. Because BASDAI does not include CRP or ESR, the response definition is not mathematically linked to the laboratory components of CALLY. However, BASDAI is entirely patient reported, and symptoms such as pain and fatigue may not always reflect inflammatory activity alone. Current recommendations therefore emphasize comprehensive assessment of disease activity rather than reliance on a single measure [32].

The recalculated CALLY index showed moderate apparent discrimination between responders and nonresponders (AUC = 0.763), without a statistically significant difference from CRP. Discrimination describes how well a marker separates outcome groups; it does not, by itself, establish accurate individual risk prediction or clinical utility. The ROC-derived Youden-optimal cutoff of 0.358 was selected from the study data, and its sensitivity and specificity may therefore be optimistic. The adjusted spline model yielded an optimism-corrected AUC of 0.753 and calibration slope of 0.853 after bootstrap internal validation, indicating some overfitting. Neither the cutoff nor the model has been externally validated, and neither should guide treatment selection.

The association between baseline CALLY and BASDAI50 achievement persisted in the multivariable model incorporating age, sex, and initial BASDAI scores. This finding suggests that the relationship was not entirely attributable to differences in these measured baseline variables. However, given the observational and retrospective nature of the study, neither causality nor the absence of residual confounding can be established. Earlier investigations have reported associations between therapeutic outcomes and various patient-related factors, including age, disease duration, functional impairment, CRP concentrations, HLA-B27 positivity, and the degree of systemic inflammation. Nevertheless, the strength and direction of these relationships have differed across study populations and definitions of treatment response [33,34]. This heterogeneity supports considering CALLY as a candidate marker rather than an independent determinant of treatment response.

Several limitations warrant consideration. The retrospective, single-center design restricts generalizability and does not support causal inference. Treatment regimens were heterogeneous, and small subgroup sizes limited treatment-specific analyses. BASDAI50 captures patient-reported improvement rather than an objective measure of inflammatory response. The ROC-derived cutoff was not externally validated, and three months of follow-up cannot establish longer-term outcomes.

Notwithstanding these limitations, several aspects of the study deserve emphasis. The analysis involved 209 patients and focused on a predefined clinical outcome assessed after three months of treatment, extending beyond the cross-sectional evaluation of disease activity. Furthermore, the relationship between baseline CALLY and therapeutic response was examined using complementary statistical approaches, including between-group comparisons, correlation analyses, multivariable logistic regression, and ROC curve analysis. Moreover, the index is based entirely on inexpensive laboratory parameters routinely available in clinical practice. The relatively small number of nonresponders (*n* = 42) also limited the number of covariates that could be included in the multivariable model. Although the sensitivity analysis excluding JAK inhibitor recipients yielded consistent findings, residual confounding related to disease duration, treatment selection, and extra-articular manifestations cannot be excluded. Furthermore, the possibility of a nonlinear association between CALLY and BASDAI50 response should be considered when interpreting the adjusted odds ratio from the primary logistic regression model. In addition, multiple exploratory comparisons were performed without formal adjustment for multiplicity. Consequently, findings with borderline *p*-values, particularly the weak correlation between lymphocyte count and BASDAI improvement (ρ = 0.138, *p* = 0.047), may represent chance associations and require confirmation in independent cohorts. Furthermore, the exclusion of patients with gastrointestinal diseases, including inflammatory bowel disease, may limit the generalizability of our findings to the broader ankylosing spondylitis population. As concomitant inflammatory bowel disease may influence treatment selection and clinical outcomes, the predictive performance of CALLY in this subgroup remains uncertain.

Baseline CALLY was nonlinearly associated with three-month BASDAI50 response in this retrospective cohort. Although it showed moderate apparent discrimination, internal validation indicated some optimism, and CALLY did not outperform CRP or add demonstrable value beyond it. An observed association and discrimination within a study sample should not be interpreted as validated individual-level prediction or clinical utility. Independent external validation is needed before clinical use can be considered.

## Figures and Tables

**Figure 1:**
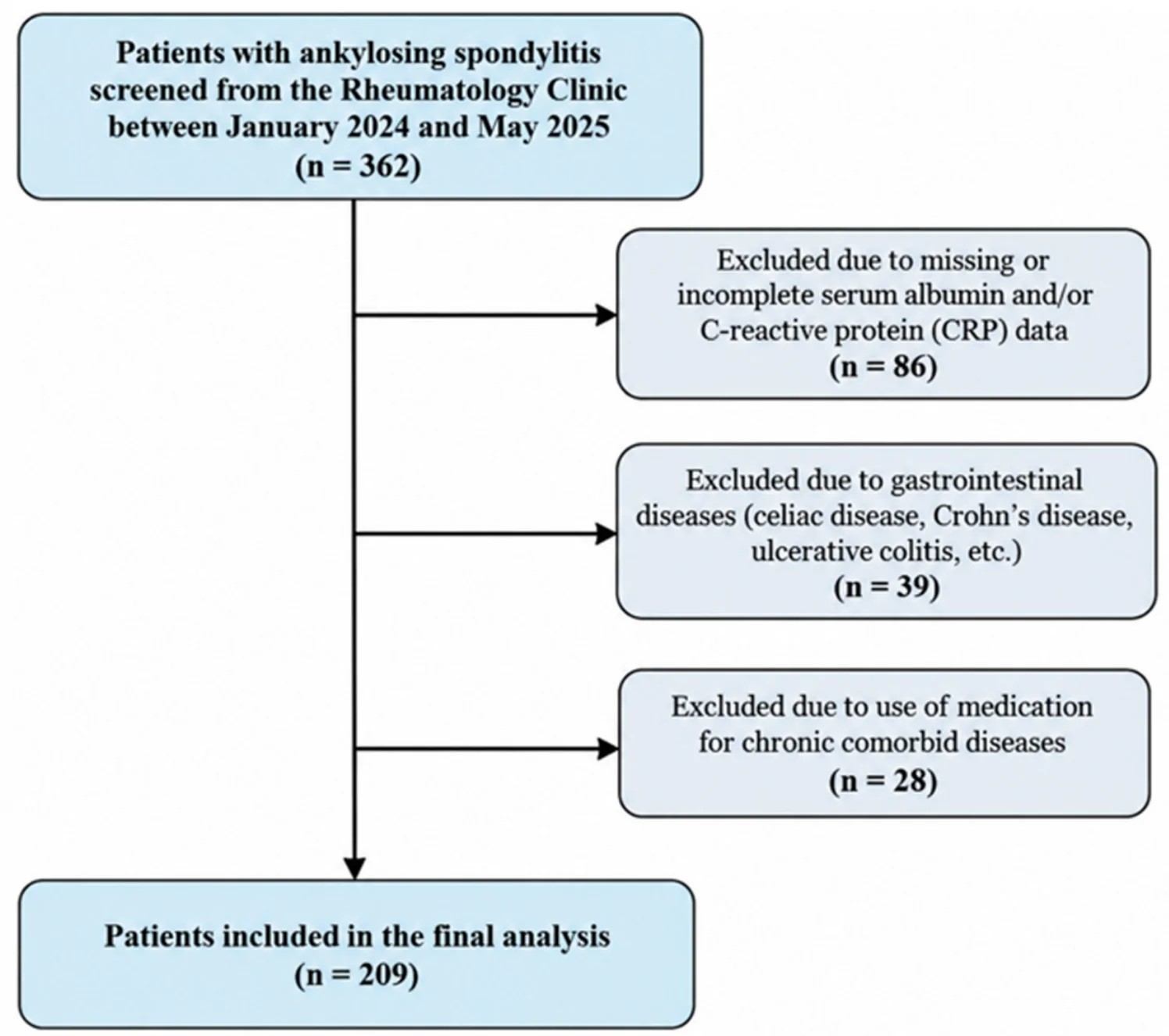
Flowchart of patient selection. A total of 362 patients with ankylosing spondylitis were screened. Patients with missing or incomplete serum albumin and/or CRP data (*n* = 86), those with gastrointestinal diseases (*n* = 39), and those using medication for chronic comorbid diseases (*n* = 28) were excluded. Finally, 209 patients were included in the analysis.

**Figure 2:**
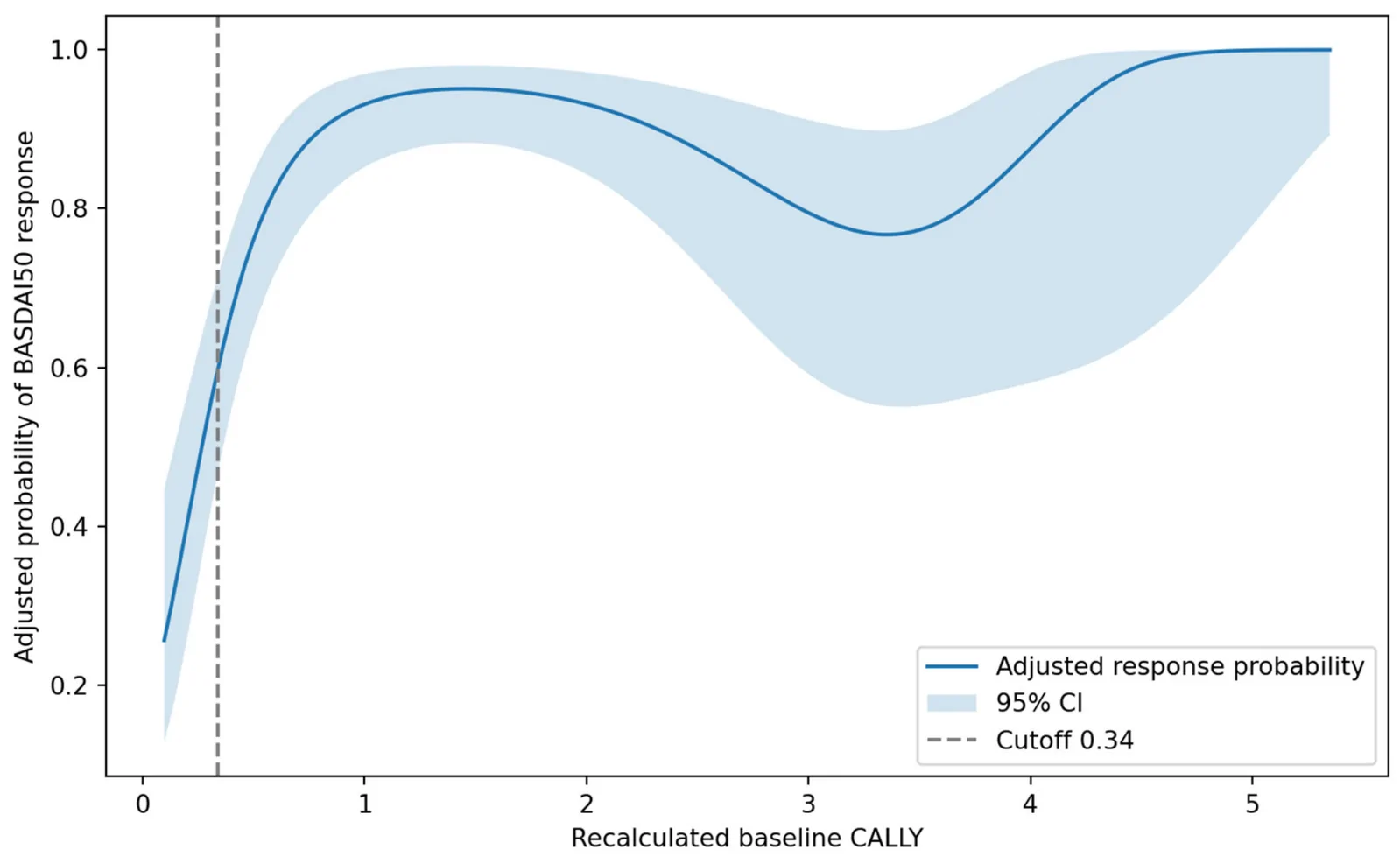
Covariate-adjusted cubic B-spline relationship between baseline CALLY and BASDAI50 response (95% confidence interval).

**Figure 3:**
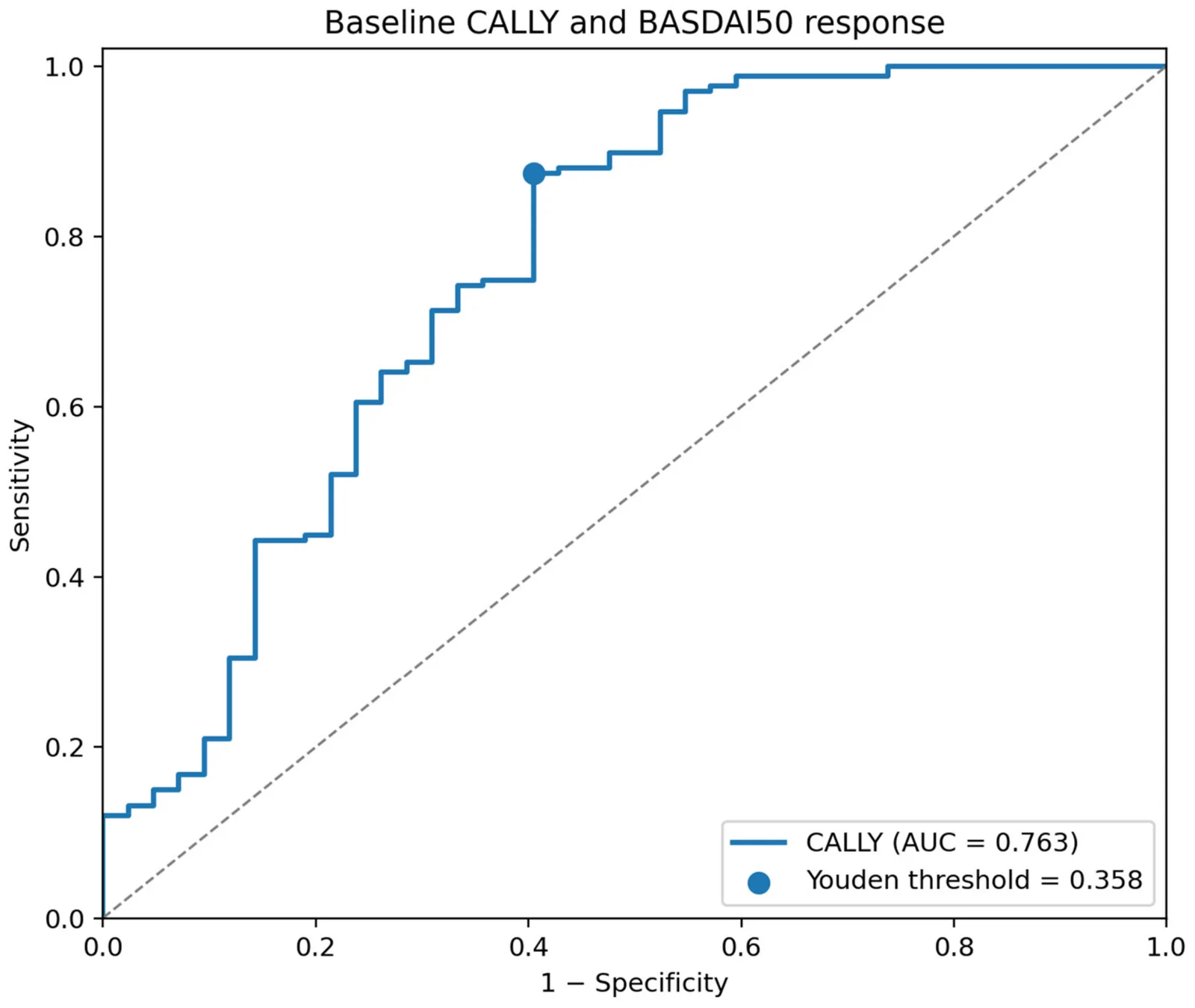
ROC curve for baseline recalculated CALLY and BASDAI50 response at month 3. AUC = 0.763 (95% stratified bootstrap CI: 0.671–0.852). The Youden-optimal threshold was 0.358, with sensitivity 87.4% and specificity 59.5%. The diagonal line represents chance discrimination.

## Data Availability

The datasets analyzed in this study may be obtained from the corresponding author upon reasonable request. Access to these data is subject to applicable ethical requirements and institutional regulations.

## Supplementary Materials

The following supporting information can be downloaded at: https://www.mdpi.com/article/10.3390/jcm15218446/s1, Table S1: Clinical and laboratory characteristics according to BASDAI50 response at month 3; Table S2: Correlations between baseline laboratory parameters and percentage improvement in BASDAI at month 3; Table S3: Multivariable logistic regression analysis for BASDAI50 response at month 3; Table S4: ROC analysis of baseline CALLY for BASDAI50 response at month 3.
