# Supplementary material for: Association of the CALLY Index with Short-Term Treatment Response in Ankylosing Spondylitis
Source: J Clin Med. 2026 Oct 30;15(21):8446. doi: 10.3390/jcm15218446 (PMC13764324; doi:10.3390/jcm15218446)
Supplement: Supplementary file 1 [file jcm-15-08446-s001.zip › jcm-4651815-supplementary.pdf]

Table S1. Clinical and laboratory characteristics according to BASDAI50 response at month 3

Variable	Non-responder (n=42)	BASDAI50 responder (n=167)	p
Age, years	43.81 ± 10.83	42.69 ± 11.71	0.574
Male sex, n (%)	32 (76.2)	108 (64.7)	0.156
Baseline BASDAI	6.55 ± 0.86	6.49 ± 0.77	0.681
Month-3 BASDAI	4.11 ± 0.74	2.39 ± 0.53	<0.001
Absolute BASDAI reduction	2.44 ± 0.76	4.10 ± 0.72	
BASDAI improvement (%)	37.07 ± 9.65	63.08 ± 7.23	
CALLY index	0.32 (0.16–0.81)	1.12 (0.54–2.75)	<0.001
CRP, mg/dL	2.89 (0.98–4.38)	0.92 (0.33–1.65)	<0.001
ESR, mm/h	32.5 (17.65–55.0)	19.0 (10.0–32.0)	<0.001
Lymphocyte, /μL	2125 (1625–2705)	2295 (1837.5–2910)	0.064
Hemoglobin, g/dL	13.25 ± 2.08	14.11 ± 1.76	0.007
WBC, $\times 10^3/\mu L$	8.76 ± 2.90	8.22 ± 2.11	0.258
Platelet, $\times 10^3/\mu L$	326.48 ± 106.49	286.06 ± 70.63	0.024
Albumin, g/dL	4.14 ± 0.52	4.29 ± 0.39	0.086

Data are presented as mean ± standard deviation, median (interquartile range), or number (percentage), as appropriate. BASDAI, Bath Ankylosing Spondylitis Disease Activity Index; CALLY, C-reactive protein–albumin–lymphocyte index; CRP, C-reactive protein; ESR, erythrocyte sedimentation rate; WBC, white blood cell count.

Table S2. Correlations between baseline laboratory parameters and percentage improvement in BASDAI at month 3

Baseline parameter	Spearman's ρ	p
CALLY	0.316	<0.001
CRP	−0.314	<0.001
ESR	−0.243	<0.001
Albumin	0.087	0.212
Lymphocyte	0.138	0.047
Hemoglobin	0.204	0.003
Platelet	−0.132	0.057

Percentage improvement in BASDAI was calculated as [(baseline BASDAI − month-3 BASDAI) / baseline BASDAI] × 100; therefore, higher positive values indicate greater improvement. Correlations were assessed using Spearman's rank correlation coefficient. BASDAI, Bath Ankylosing Spondylitis Disease Activity Index; CALLY, C-reactive protein–albumin–lymphocyte index; CRP, C-reactive protein; ESR, erythrocyte sedimentation rate.

Table S3. Multivariable logistic regression analysis for BASDAI50 response at month 3

Variable	OR	95% CI	p
CALLY	1.925	1.297–2.857	0.001
Age	0.996	0.964–1.028	0.786
Female sex	1.633	0.715–3.731	0.244
Baseline BASDAI	0.981	0.629–1.530	0.931

The dependent variable was BASDAI50 response at month 3 (responder = 1, non-responder = 0). The linear logistic model included age, female sex, baseline BASDAI, and recalculated CALLY (n = 209). The CALLY OR is per one-unit increase under the linear model; the spline analysis demonstrated significant nonlinearity. OR, odds ratio; CI, confidence interval.

Table S4. ROC analysis of baseline CALLY for BASDAI50 response at month 3

Variable	AUC	95% CI	Optimal cut-off	Sensitivity	Specificity	p
CALLY	0.763	0.671–0.852	0.358	87.4%	59.5%	<0.001

The optimal cut-off was selected by the Youden index in the same cohort; estimates are apparent and require external validation. The 95% AUC CI was estimated by 5,000 outcome-stratified bootstrap resamples. At the 0.358 threshold: true positives = 146, false negatives = 21, true negatives = 25, false positives = 17 (n = 209). Sensitivity was 87.4% (95% Wilson CI: 81.5–91.6%) and specificity was 59.5% (95% Wilson CI: 44.5–73.0%). AUC, area under the ROC curve; CI, confidence interval.
